# Stabilization of the obesity epidemic and increasing thinness in children in Caribbean Bonaire

**DOI:** 10.1186/s12887-018-1146-3

**Published:** 2018-05-17

**Authors:** Joana Kist-van Holthe, Tirza Blom, Laura Melchers, Alcira Janga-Jansen, Tahirih van Kanten, Marian Luinstra-Passchier, Teatske Altenburg, Remy HiraSing, Mai Chinapaw

**Affiliations:** 1 Department of Public and Occupational Health, Amsterdam Public Health Research Institute, VU Medical Center, Amsterdam, the Netherlands; 2 Department of Public Health, Openbaar Lichaam Bonaire, Bonaire, the Netherlands

**Keywords:** Obesity, Children, Overweight, Thinness, Caribbean, Lifestyle, Diet, Physical activity, Sedentary behavior

## Abstract

**Background:**

In 2008, the prevalence of overweight and obesity among children in Bonaire was twice as high as the prevalence in northern Europe but comparable to that of other Caribbean islands and the United States. The aim of this study was to examine change in the body mass index status of children in Bonaire and report children’s energy balance-related behaviours (EBRB) in 2015.

**Methods:**

Two school-based cross sectional surveys of children age 4-14 years were conducted in 2008 and 2015. Height (m) and weight (kg) were measured, body mass index (BMI) calculated and children’s BMI categorised according to the International Obesity Task Force criteria. In 2015, children age 10-14 years completed a questionnaire on EBRB and responses were compared between non-overweight/obese children and overweight/obese children.

**Results:**

In total 2117 children age 4-14 years participated (92.4% response rate). The prevalence of thinness significantly increased between 2008 and 2015 (adjusted OR 1.5 95% CI: 1.2-1.8). There were no other significant differences in children’s weight status between survey years. One quarter of children (25.4%) were overweight/obese in 2015. There were no significant differences in EBRB between non-overweight/obese and overweight/obese children in 2015. Few children met recommendations for EBRB.

**Conclusions:**

The prevalence of overweight/obesity in children in Bonaire did not significantly change between 2008 and 2015 and remained high. However, the prevalence of thinness has significantly increased. Interventions to improve children’s current EBRB are required.

**Electronic supplementary material:**

The online version of this article (10.1186/s12887-018-1146-3) contains supplementary material, which is available to authorized users.

## Background

The prevalence of childhood overweight and obesity has rapidly increased worldwide in recent decades [1]. Children are developing obesity at a younger age, and the degree of obesity among children is increasing. More importantly, overweight and obesity “track” into adulthood, implicating a 2-15 times higher chance of becoming an obese adult when being obese during childhood [2]. Overweight and obesity can lead to important health issues, e.g., type 2 diabetes, cardiovascular disease, fatty liver disease, psychological problems and musculoskeletal disorders [3, 4]. In Bonaire, an island in the Caribbean and part of the Netherlands, 35% of the adult population in 2013 was overweight, 25% was obese, and 8 % had type 2 diabetes [5, 6].

In 2008, we first studied the prevalence of overweight and obesity in children in Bonaire. At that time, 14.3% of the boys (aged 4-14 years) and 18.6% of the girls were overweight, and 9.8% of the boys and 13.0% of the girls were obese [7]. The prevalence of overweight is approximately twice as high as that in countries in northern Europe but is comparable to that of other islands in the Caribbean and the United States [7]. Thinness was observed in 10.4% of boys and 8.5% of girls.

The aim of this study was to obtain an update on the body mass index (BMI) status of children in Bonaire. In addition, we studied energy balance-related behaviors to obtain clues for the tailored prevention of obesity.

## Methods

### General

All schools in Bonaire (nine elementary schools and one high school) were asked to participate in a cross-sectional study from March to May 2015. Because attending school is mandatory up to the age of 16 years, the study population comprised all school-attending children. No incentives were given. One small elementary school (*n* = 103 children) declined to participate for unknown reasons. Weight and height were measured in all 4- to 14-year-old children, and children 10-14 years of age were asked to complete a questionnaire pertaining to energy balance-related behaviors. The same study design was used as that in a previous study in children in Bonaire in 2008 [7]. The VU University Medical Ethical Committee has revised the study protocol and concluded that the study does not fall within the scope of the Medical Research Involving Human Subjects Act. Parental permission for participation in the study was given by means of passive informed consent. All parents received a letter informing them of the study and that they could opt out by filling in a form and sending it back to the school.

### Anthropometric measurements

Two trained assistants performed the anthropometric measurements at the schools. Body weight was measured to the nearest 0.1 kg using a digital scale (Seca 877®, Hamburg, Germany). Because children were weighed in summer clothes without shoes, the following correction for clothing was performed: − 300 g for children 4-6 years; − 600 g for children 7-9 years; − 800 g for those aged 10-11 years; and − 1000 g for children ≥12 years. Height was measured to the nearest 0.1 cm without shoes using a microtoise (Seca 206®, Hamburg, Germany). BMI was calculated as weight (kg) /height (m^2^) and classified as thinness, normal weight, overweight, obesity and morbid obesity according to the cut points of the International Obesity Task Force [8].

### Energy balance-related behavior questionnaire

Children ≥10 years of age were asked to complete a questionnaire during class pertaining to energy balance-related behaviors based on a previously validated questionnaire among European children 10-12 years of age [9]. The questionnaire was adapted to the Caribbean situation so that the children could easily understand the questions (Additional file 1, energy balance-related questionnaire), e.g., by giving examples of frequently used (brand) names of sweetened drinks and local vegetables. The questionnaire was translated into Papiamento and Dutch. The results were compared to recommendations for children for fruit (≥ 2 pieces per day) and vegetable intake (≥ 150-200 g per day equal to 3-4 ladles per day) [10], physical activity (≥ 1 h per day of moderate- to vigorous-intensity physical activity) [11], television / media time (maximum 2 h per day) [12] and sleep [13, 14]. The recommended sleep duration for children 10 years of age is ≥10.5 h; for 11- and 12-year-olds, the recommendation is ≥10 h, and for 13- and 14-year-olds, the recommendation is ≥9.5 h [13, 14].

### Statistics

Analyses were performed with SPSS 22.0 (IBM®, New York, USA). Differences in BMI status (thinness, overweight, obesity and morbid obesity) in 2015 compared to 2008 were tested with logistic regression. Analyses were adjusted for age, gender and clustering of data within schools. In addition, we checked whether differences in BMI status between 2008 and 2015 differed by gender and age group by including an interaction term. As gender and age group were no effect modifiers only the overall Odds ratios are presented.

The difference in energy balance-related behaviors between non-overweight and overweight children was tested with the Pearson Chi-squared test (categorical variables), t-test (continuous variables with normal distribution) and the Mann-Whitney test (continuous variables with skewed distribution). Because of the large number of tests, a *p*-value < 0.01 was considered statistically significant.

## Results

### BMI status

**Fig. 1:**
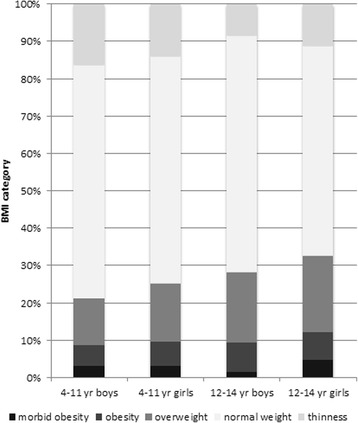
Body mass index (BMI) categories in 4- to 11 and 12- 14-year-old boys and girls in in 2015 in Bonaire

A total of 2117 of 2291 (92.4%) 4- to 14-year-old children (1070 (50.5%) boys) in Bonaire participated in the study. Anthropometric measurements were not performed in 174 children (7.6%) because these children were not at school on the day of the measurements or because the parents did not want their child to participate in the study. Table 1 presents the prevalence (%) of the body mass index (BMI) status of children in Bonaire in 2008 and 2015. The prevalence of thinness, normal weight, overweight, obesity and morbid obesity in children was 13.7, 61.0, 15.5, 6.4 and 3.5%, respectively. In 2015, the prevalence of thinness was higher in 4-11 than 12-14 year olds (15.1 versus 9.7%), while the prevalence of overweight and obesity were higher in 12-14 than 4-11 year olds (19.6 versus 14% and 7.7 versus 5.9%, respectively). BMI status was similar in boys and girls (Fig. 1). The prevalence of thinness in primary school children in Bonaire was significantly higher in 2015 than in 2008 AOR 1.5 (95%CI: 1.2-1.8). The prevalence of overweight, obesity or morbid obesity was similar in 2015 and 2008 AOR 1.1 (95% CI: 0.9-1.3), 1.0 (95% CI:0.7-1.2) and 1.0 (95% CI: 0.7-1.4), respectively.

**Table 1:** Prevalence (%) of the body mass index (BMI) status of children in Bonaire in 2008 and 2015

		Boys		Girls		All	
Age group	BMI status	2008	2015	2008	2015	2008	2015
		%	%	%	%	%	%
4-11 years	Thinness	11.4	16.3	9.6	13.8	10.5	15.1
	Normal weight	66.8	62.2	59.7	60.8	63.4	61.5
	Overweight	12.4	12.5	18.2	15.6	15.2	14.0
	Obesity	5.8	5.5	7.6	6.3	6.7	5.9
	Morbid obesity	3.6	3.5	4.8	3.5	4.2	3.5
	Total	100	100	100	100	100	100
		*N* = 678	*N* = 793	*N* = 643	*N* = 768	*N* = 1330	*N* = 1561
12-14 years	Thinness	8.0	8.3	6.1	11.1	7.0	9.7
	Normal weight	61.8	63.2	60.4	55.9	61.1	59.5
	Overweight	19.5	18.8	19.5	20.4	19.5	19.6
	Obesity	7.3	7.9	10.9	7.5	9.2	7.7
	Morbid obesity	3.4	1.8	3.1	5.0	3.2	3.4
	Total	100	100	100	100	100	100
		*N* = 262	*N* = 277	*N* = 293	*N* = 279	*N* = 555	*N* = 556
Total		100 *N* = 949	100 *N* = 1070	100 *N* = 936	100 *N* = 1047	100 *N* = 1885	100 *N* = 2117

### Energy balance-related behavior questionnaire

A total of 768 (75%) of 1029 10- to 14-year-old children in Bonaire completed the questionnaire. The response rate varied per question from 583 (57%) to 768 (75%). The percentage of missing questionnaires included children who were not at school on the day of the survey (7.9%). Table 2 denotes the results of the questionnaire pertaining to energy balance-related behaviors. There were no significant differences between non-overweight and overweight children in self-reported dietary patterns, physical activity, screen time or sleep duration. A large number of the children in Bonaire did not fulfill the recommendations for a healthy lifestyle; for example, only 50% of the children attained the recommended daily intake of vegetables, and only 37% met the recommendations for fruit intake.

**Table 2:** Energy balance-related behaviors among 10- to 14-year-old children (N = 768) in 2015 in Bonaire

Energy balance-related behaviors (Percentage complying with recommendations)	overweight or obese children	non-overweight children	*p*-value
	*N* = 247	*N* = 521	
Nutrition			
Ate breakfast this morning	71.7	75.4	0.26
Eats two or more pieces of fresh fruit/day	37.4	37.4	0.97
Eats two or more portions of vegetables/day	50.2	50.3	0.97
Drinks one or less sweetened drinks/week	17.9	15.0	0.28
Physical activity/screen behavior			
Participates one or more times / week in organized sports	82.8	75.7	0.03
Participates in one or more hours of physical activity on a school day	68.0	67.8	0.94
Watches two or more hours of television on a school day	73.1	72.8	0.93
Uses the computer for two or more hours on a school day	62.8	65.9	0.39
Uses active transportation to school	21.1	23.8	0.40
Sleep duration			
10-year-old children, 10.5 h or more	94.4	88.6	0.33
11- & 12-year-old children, 10 h or more	73.8	66.1	0.17
13- & 14-year-old children, 9.5 h or more	88.2	86.0	0.65
Mean (SD) sleep duration (h)	8.7 (1.37)	8.7 (1.36)	0.98

The response rate per question varied from 583 (57%) to 768 (75%). Fruit and vegetable consumption, physical activity, television and computer time pertain to the previous day and breakfast to the morning of the survey; active transportation to school includes walking or biking. In view of the many parameters tested, *P* < 0.01 is considered significant

## Discussion

Although the prevalence of overweight and obesity in children in Bonaire in 2015 was high, it was similar to that of 2008. This is a promising development. Two recent large-scale studies on childhood obesity, with data from the United States, China, Australia, New Zealand and Europe, demonstrated a similar stabilization in the prevalence of overweight and obesity [15, 16]. To the best of our knowledge, this is the first study examining the prevalence of overweight and obesity over time in Caribbean children.

Remarkably, thinness was noted in nearly 14% of the children. This is more than two times the prevalence of thinness in Puerto Rico (5.7%) [17]. Equally, using the International Obesity Task Force definition of thinness in 5- to 14-year-old children in Guadeloupe, Martinique, French Guiana and French Polynesia, thinness varied from 12.7 to 19.4% in girls and from 4.8 to 13.3% in boys [18]. Our study was not designed to provide clues as to why the prevalence of thinness – especially that in young children (4 and 5 years of age) - in Bonaire is high and has increased since 2008.

There were no significant differences between non-overweight and overweight children in Bonaire in terms of self-reported dietary patterns, physical activity, screen time or sleep duration. However, a large number of the children in Bonaire did not fulfill the recommendations for a healthy lifestyle. Only 50% of children attained the recommended daily intake for vegetables (≥ 2 portions), and 37% met the recommendations for fruit (≥ 2 pieces). High prices of fresh fruit and vegetables may play a role. Nearly all fresh fruits and vegetables are imported into Bonaire. However, compared with 10-12-year-old European children, the percentage of children in Bonaire complying with the recommendations was similar for fruit (European children 15 to 34%) and higher for vegetables (European children 15 to 32%) [19]. Approximately 25% of the children in Bonaire skipped breakfast, which resembles data from 10- to 12-year-old children in Europe, where skipping breakfast at least once a week varied from 14% (Spain) to 51% (Slovenia) [20]. In the same study, children slept (parent-reported) a mean of 9.2 h a night, varying from 8.7 h in Greek boys to 9.7 h in Belgian girls [20]. Compared with children in Europe, sleep duration in children in Bonaire was relatively short (8.7 h).

Two-thirds of children in Bonaire reported to engage in the recommended ≥1 h of moderate-to-vigorous physical activity per day. This percentage is higher than found in a large cross-sectional survey of almost 220,000 youths (11–15 years) from 42 participating countries of the 2013–2014 Health Behaviour in School-Aged Children Study. The percentage of study participants that reported to engage in moderate-to-vigorous physical activity for 60 or more minutes per day ranged from 8 to 47% for boys and from 5 to 34% for girls [21]. More than one-quarter of the children in Bonaire watched more than the advised maximum of 2 hours of television on a school day. This is less than the proportion of 10-12-year-old European children, of whom approximately 38% reported to watch more than 2 hours of television per day [22].

The strength of this study is the high response rate: nearly all (92.4%) 4- to 14-year-old children in Bonaire participated in the study. In addition, all anthropometric measurements were performed by the same trained research assistants. Furthermore, the same study design was used in the previous study in Bonaire (2008) by the same research group, thereby justifying comparison of the anthropometric data [7]. However, the content and delivery of the questionnaire in 2015 differed from that in 2008, impeding the comparison of energy balance-related behavior.

There are some weaknesses in the study. As some children who seemed overweight were not allowed to participate in the study by their parents, the prevalence of overweight and obesity may be slightly underestimated. However, the same phenomenon applied in our previous study in 2008 [7]. A limitation of the study is that psychometric testing was not done after adaptation of the questionnaire to the local situation in Bonaire. However, the adaptation only included providing cultural appropriate examples of fruits, vegetables and drinks.

Another weakness is that approximately one-quarter of the questions from the questionnaire were not completed. Furthermore, one can debate as to how correct children are in estimating the quantity of their food and drink intake, as well as their physical activity, screen- and sleep time [23–25]. To try and obtain as many reliable answers as possible, the questionnaire asked to recall intake at a certain recent moment in time (e.g., fruit and vegetable intake the day before or breakfast consumption the morning of the questionnaire). However, a three-day prospective food frequency diet may have given a more reliable overview of dietary intake. Additionally, it is possible that children gave socially acceptable answers; this may especially apply to overweight and obese children [26].

## Conclusions

The prevalence of overweight and obesity among children in Bonaire remains high. However, it is a promising development that, compared with 2008, the prevalence of overweight and obesity in children has stabilized. Conversely, the prevalence of thinness, especially in the youngest children, was high. More studies are needed to clarify the cause of thinness among (young) children in Bonaire.

Self-reported energy balance-related behaviors were not significantly different between non-overweight and overweight children. However, a large number of children in Bonaire did not fulfill the recommendations for a healthy lifestyle. This knowledge can be used as a basis for governmental health policy measures in schools (e.g., providing healthy food choices and free water in school canteens, stimulating active transport and physical activity options during recess and after school) and in the community as a whole (e.g., promoting affordable locally grown fruit and vegetables, access to affordable sport participation, and safe neighborhoods and roads).

## Additional file

Additional file 1: Questionnaire Bonaire 2015. Energy balance-related questionnaire (DOCX 21 kb)
