# Supplementary material for: Stabilization of the obesity epidemic and increasing thinness in children in Caribbean Bonaire
Source: BMC Pediatr. 2018 May 17;18:168. doi: 10.1186/s12887-018-1146-3 (PMC5960189; doi:10.1186/s12887-018-1146-3)
Supplement: Supplementary file 1 — Questionnaire Bonaire 2015. Energy balance-related questionnaire (DOCX 21 kb) [file 12887_2018_1146_MOESM1_ESM.docx]

**Supplemental file**

**Questionnaire Bonaire 2015**

**Age** ..........................................................................................................

**Gender** O boy O girl

**School**  ……………………………………...………………………………

1. Did you have breakfast this morning?

O yes

O no

2. How many vegetables did you eat yesterday (for example: pumpkin, spinach, cabbage, tomatoes, pepper bell, carrot etcetera (potatoes and beans do not count as vegetables))?

O none

O 1 ladle

O 2 ladles

O 3 ladles

O 4 or more ladles

3. How much fruit did you eat yesterday?

O none

O 1 piece / portion

O 2 pieces / portions

O 3 pieces / portions

O 4 or more pieces / portions

The next question is about sweetened drinks, for example:


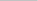


| **Fruit drinks** |   **Powder- drinks** | **Soft drinks** | **Milk or yogurt drinks** |   **Sport- drinks** |   **Lemonade syrup** |    **Energy- drinks** |   **Other** |
| --- | --- | --- | --- | --- | --- | --- | --- |
| Gloria | Koolaid | Sinas | Yogurtdrink | AA | Roosvice | Red bull | Tea with sugar |
| Rica | Tang | Limonade | Yogo Yogo | Extran | Sunquick | Monster | Icetea |
| Del Prado |  | Cola | Yokidrink | Isostar | Ranja |  |  |
| Multivitamine |  | Sisi | Chocomelk | Aquarius |  |  |  |
| Dubbeldrank |  | Dr. Pepper | Fristi |  |  |  |  |
| Caprisun |  | Fernandez | Chicha |  |  |  |  |
| Dubbel fris |  | Faygo |  |  |  |  |  |

4. How many times per week do you drink sweetened drinks?

O never

O less than 1 time per week

O 1 time per week

O 2-4 days per week

O 5-6 days per week

O every day, 1 time per day

O every day, more than 1 time per day

5. How many times per week do you participate in organized sports?

O never

O 1 – 2 / week

O 3 – 4 / week

O 5 – 6 / week

6. How many hours per day do you play or exercise or participate in sports outside on a school day (for example, walking, biking, swimming)

O none

O less than 30 minutes per day

O 1 hour / day

O 1.5 hour / day

O 2 hours / day

O 2.5 hours / day

O 3 hours / day

O 3.5 hours / day

O more than 4 hours / day

7. How many hours per day do you watch television on a school day?

O none

O less than 30 minutes / day

O 1 hour / day

O 1.5 hour / day

O 2 hours / day

O 2.5 hours / day

O 3 hours / day

O 3.5 hours / day

O 4 hours / day

O 4.5 hours / day

O more than 5 hours / day

8. How many hours per day do you play games on the computer, games console, tablet, smartphone, or television or do you use the computer to chat online, for internet, email etcetera.

O none

O less than 30 minutes per day

O 1 hour / day

O 1.5 hour / day

O 2 hours / day

O 2.5 hours / day

O 3 hours / day

O 3.5 hours / day

O 4 hours / day

O 4.5 hours / day

O more than 5 hours / day

9. How did you get to school today?

O walking

O by school bus

O by bicycle

O by car

O by public transportation

10. At what time do you go to bed at night on a school day?

O around 7 o’clock

O around 8 o’clock

O around 9 o’clock

O around 10 o’clock

O around 11 o’clock

O around or after 12 o’clock

O it varies

11. At what time do you get up in the morning on a school day?

O around 4 o’clock

O around 5 o’clock

O around 6 o’clock

O around 7 o’clock

O around 8 o’clock

O it varies
